# Minimally Invasive Extracorporeal Technologies Perspective in Pediatric Cardiac Surgery

**DOI:** 10.21470/1678-9741-2020-0471

**Published:** 2022

**Authors:** Ignazio Condello

**Affiliations:** 1 Department of Cardiac Surgery, Anthea Hospital, GVM Care & Research, Bari, Italy. E-mail: ignicondello@hotmail.it

Minimally invasive extracorporeal technologies (MiECT) are in great expansion in the literature and have an impact in adult cardiac surgery, however, a future challenge will be to expand MiECT to pediatric cardiac surgery. In the context of pediatric MiECT, there are not enough studies in the literature on this topic, which seems a paradox because the optimizations in cardiopulmonary bypass (CPB) are the essence on literature and practice in the pediatric approach during cardiac surgery. In pediatric cardiac surgery, body weight-adjusted miniaturized CPB circuits within a comprehensive blood-sparing approach can reduce transfusion requirements. Haemodilution resulting from mixing the patient's blood with a CPB crystalloid solution may be reduced to the extent that asanguineous priming becomes possible ^[1]^. Therefore, we adopted asanguineous priming in our clinical routine ^[2]^. However, optimizations in the traditional circuit, if on the one hand reduce the consumption of blood products, on the other, require the use of vacuum-assisted venous drainage (VAVD). VAVD is essential to reduce the length of the circuit, to place the oxygenating system and the venous reservoir at the child patient's height and to optimize venous return. We read with high interest the article “Impact of Vacuum-Assisted Venous Drainage on Forward Flow in Simulated Pediatric Cardiopulmonary Bypass Circuits Utilizing a Centrifugal Arterial Pump Head”, by Guimarães et al. ^[3]^. The authors analyze the impact of VAVD on arterial pump flow in a simulated pediatric CPB circuit utilizing a centrifugal pump (CP) with an external arterial filter. The study concluded that the use of VAVD reduces arterial flow when a CP is used as the main arterial pump. The reduction in forward arterial flow increases as the vacuum level increases. The loss of forward flow is further reduced when the arterial filter purge line is kept in the recommended open position. The use of a minimally invasive extracorporeal circulation (MiECC) system would allow, in this context, to maximize the benefits already obtained with the optimized CPB, removing and eliminating the use of VAVD, roller pumps and blood-air interface (BAI). In a study by Kadner et al. ^[3]^, a total of 38 pediatric patients underwent surgical interventions for a variety of congenital heart diseases with MiECC. In these patients, MiECC perfusion was successfully performed in all patients (100%). Median patient age was 9.5 months (range 0.2-176 months) with a median weight of 8.1 kg (range 2.3-49 kg). For both MiECC types, no system-related technical complications were found. MiECT can be performed using standard techniques for closed and open cardiac procedures for the correction of a variety of malformations in neonates and children with good results and uneventful postoperative course.
